# Investigation of Rutting Performance in Geogrid-Reinforced Asphalt by Penetration Test

**DOI:** 10.3390/ma16227221

**Published:** 2023-11-18

**Authors:** Sheng-Lin Wang, Danrong Wang, Susan Tighe, Sam Bhat, Shunde Yin

**Affiliations:** 1 Department of Civil and Environmental Engineering, Faculty of Engineering, University of Waterloo, Waterloo, ON N2L 3G1, Canada; peggie.wang@uwaterloo.ca; 2 Department of Civil Engineering, McMaster University, Hamilton, ON L8S 4L8, Canada; tighes1@mcmaster.ca; 3 Titan Environmental Containment Ltd., Ile des Chenes, MB R0A 0T1, Canada; sam@titanenviro.ca

**Keywords:** geosynthetics, reinforcement, rutting, penetration test, flow number

## Abstract

Permanent deformation, or rutting, is one of several critical distresses in flexible pavements. This paper introduced a novel experimental method, a penetration test, for asphalt mixtures to quantify the effects of glass fibre geogrids embedded in asphalt under repeated loading. It was found that the evolution of permanent deformation (*ε_p_*) and its strain rate have three clearly identifiable stages. It was also observed that the presence of the geogrid increased the flow number and the number of cycles to failure significantly compared to control samples. Some of the current *ε_p_* fitting models were found to be valid for deformation prediction under penetration. In addition, a new simple FN calculation method was also proposed based on strain rate and it showed consistent results. In particular, geogrid type “Grid10”, which has smaller aperture size (12.7 mm) had slightly better reinforcement performance regarding the rutting resistance due to its larger contact area. Overall, the test and data analysis method presented in this study could be an important reference for future investigations on geosynthetic-reinforced pavement materials.

## 1. Introduction

Permanent deformation, or rutting, is one of the critical forms of distresses in flexible pavements [1,2]. Rutting is caused by the accumulation of unrecovered deformation caused by repeated application of heavy or slow vehicle loads [3,4]. Such deformation occurs especially in warm seasons, and could be exacerbated due to frequent freeze–thaw cycles [5].

In the past few decades, the use of paving grids has been gaining interest, particularly glass fibre grids, between asphalt layers [6,7,8]. Such technology is a reliable, robust, and cost-effective solution for asphalt reinforcement as it can decrease segregation, improve load distribution, and provide structural reinforcement and lateral confinement under the traffic loading [9,10]. Currently, it is generally agreed that geogrid reinforcement increases the resistance to rutting, fatigue, and reflective cracking [1,6,11,12]; in some cases, between 85% and 150% improvement is observed compared to ordinary hot mix asphalt [13].

Various laboratory characterizing methods have been developed to quantify the effects of rutting on asphalt concrete. These include the static/dynamic creep test [1], wheel track test [2,3,10], and flow number (FN) test [14,15]. Nevertheless, very few were addressed on the investigation of rutting test of geogrid-reinforced asphalt concrete. Several studies introduced using large-scale slabs for wheel track tests [16,17,18]. It was indicated that the geogrid-reinforced asphalt has the potential to increase the rutting resistance and up to 33% decrease on the load of subgrade [10,19]. Nevertheless, there are challenges that exist in terms of the difficulties of sample preparation, shortage of experimental dataset, and the lack of appropriate test protocols. This paper focused on the preliminary investigation on the permanent deformation of geosynthetic-reinforced asphalt. A penetration test was therefore developed, which allows the dynamic penetration into a multi-layer asphalt concrete specimen with geogrid reinforcement. Such a sample preparation method and test procedure enables the quantitative analysis for the rutting resistance of geosynthetic-reinforced asphalt. In addition, this study also gives rise to the comparisons between different geosynthetic products.

## 2. Background

Rutting in asphalt mixtures generally occurs in three stages [3,14]: the primary phase (Stage I), the linear phase (Stage II), and the tertiary phase (Stage III). In Stage I, permanent strain grows rapidly, while the strain rate decreases over time. This is mainly due to the initial compaction and densification of asphalt mixture. During Stage II (the linear phase), the permanent strain increases linearly with the increase in loading cycles. Such characteristics are related to shear stress flow. Finally, in Stage III (the tertiary phase) the strain rate rapidly increases due to the shear failure and crack propagation. The flow number (FN) is considered as the number of load cycles at which the hot mix asphalt (HMA) mix enters the tertiary phase [14]. Extensive models had been generated to predict the rutting performance of asphalt mixture, especially for primary phase and linear phase [15,20,21]. However, the analysis for geogrid-reinforced asphalt mixture is rarely seen.

In order to minimize the interruption of field construction, geogrid is usually installed between the wearing course and binder course, or within different lifts of the binder course. Generally, a minimum 40 mm to 50 mm of asphalt overlay is recommended to prevent slipping effects [6]. Regarding laboratory investigation, in order to better simulate the practical conditions and to quantify the effects of geogrid reinforcement, the whole pavement structure should be tested. In other words, such an experimental study should be performed on a full-depth specimen, which includes not only asphalt layers but also the embedded geogrid. Unfortunately, there is no widely accepted sample preparation methods or test protocol for such an investigation. Consequently, the trend of permanent strain development in geogrid-reinforced asphalt was not very well identified and investigated.

Among the current rutting performance tests introduced in American Association of State Highway and Transportation Officials (AASHTO), the FN test is a relatively easy experimental protocol and could accommodate a taller specimen (higher than 10 cm). Table 1 summarizes some popular models for quantifying the rutting performance of asphalt mixture. Based on standard FN test, common models include the Francken models (Equations (1) and (2)), detailed three-stage model (Equation (3)), FNest model (Equation (4)), and nonlinear viscoelastic (NLVED) model (Equation (5)).

where, *ε_p_* = accumulated permanent strain, total strain, με;
*ε_I_*, *ε_II_* = accumulated permanent strain corresponding to the end of Stage I and Stage II, με;
*N* = number of total load repetitions;
*N_I_*, *N_II_* = number of load repetitions corresponding to the end of Stage I and Stage II, onset of next stage.

Furthermore, FN is often referred to as the number of cycles corresponding to the minimum strain rate, or the flex point [18,25]. The first step in this process is to obtain the permanent strain rate directly from the permanent accumulation data. Afterwards, the rate curve data may be smoothed (using a moving average recalculation, for example) to help determine the FN. However, challenges will be to quantitively determine the transition points (*N_I_*, *N_II_*) between different phases. Currently, for three-stage models, the deviation between predict and the experimental strain was used to determine such transition points. As soon as the deviation of prediction exceed the criterion (3% used in this study), it indicates that the stress–strain relationship has entered the next phase [23]. As for other models, it does not imply the specific method to calculate the transition point when entering the linear phase, while the transition point of entering the tertiary phase (flow number) was calculated using the proposed equations. This applies for FNest and the NLVED method.

Recently, a two-step secant method was introduced to have less data noise and reduce the number of potential FN solutions to one. This method relies on applying the secant method twice to determine the load cycle when the deformation curve changes from convex to concave [15]. The disadvantage of this method, however, is its operational complexity. In addition, such method requires data collection continuing until the accumulated permanent strain reaches 40,000 με (micro strain).

In the following sections, selected models will be validated using laboratory results from the proposed penetration test. Therefore, the development of permanent strain in geogrid–reinforced asphalt will be predicted and the FN will be calculated.

## 3. Materials and Methodology

### 3.1. Asphalt Mixture

**Figure 1:**
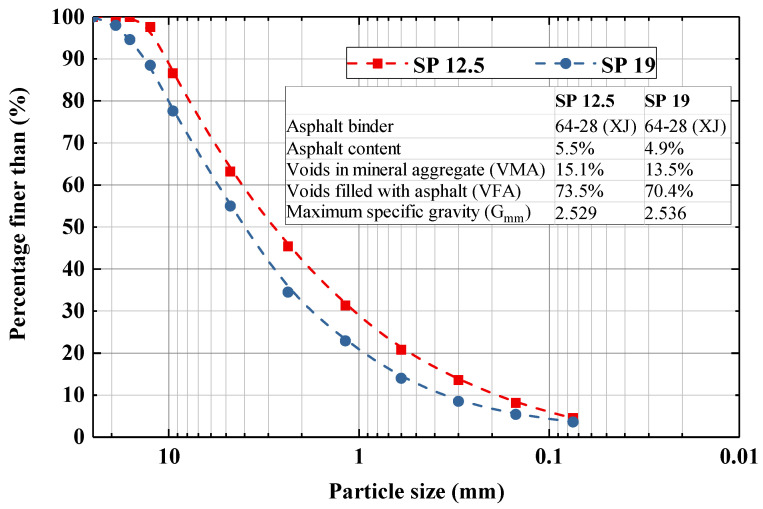
Particle size distribution for SP 12.5 and SP 19.

Superpave (SP) grades SP 12.5 and SP 19 were used for this study, acting as an asphalt wearing course, and binder course, respectively. Between them, 20% by weight of SP 19 are made of reclaimed asphalt pavement (RAP). Their particle size distributions are presented in Figure 1. The asphalt binder used for both mixes were performance grade (PG) 64-28, with asphalt content accounting for 5.5% and 4.9% for SP 12.5 and SP 19, respectively. The theoretical maximum specific gravity G_mm_ for both mixes were then characterized using the maximum relative density (MRD) test.

### 3.2. Fibreglass Geogrids

**Figure 2:**
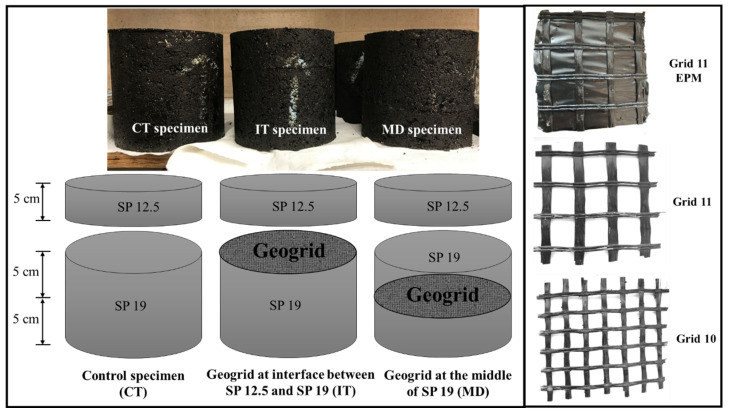
Specimen designation for penetration test.

Three types of glass fibre geogrids were used in this research, namely Grid10, Grid11, and Grid11EPM, from Titan Environmental Containment Ltd., Ile des Chênes, Canada. All the three types of geogrids are polymer coated. Bonding between geogrid and lower side asphalt is improved by applying a tack coat after the compaction of the initial layer. Among them, Grid11EPM is bonded to an engineered polymeric membrane (EPM) on top of the grid. The EPM starts to melt at 80 °C and will be a complete melt while the temperature is above 124 °C. Therefore, it created an additional adhesion to the asphalt layer above during the paving process. The physical properties for the three types of geogrids are summarized in Table 2. As it was shown, the three types of geogrids have very similar physical properties regarding the tensile strength, the secant stiffness, and the density. However, Grid10 has half the aperture size (12.7 mm × 12.7 mm) compared to those of the Grid11 and Grid11EPM (25.4 mm × 25.4 mm). Generally, Grid10 is used with fine asphalt and Grid11 is used with course asphalt in order to facilitate maximum interlocking effects. In addition, Grid10 has the same unit mass compared to Grid11, although its aperture size is smaller. It could be due to the thinner fibre width in Grid10 compared with the other two types of geogrids, as it is seen in Figure 2. On the other hand, the Grid11EPM has a heavier mass unit since it has an additional EPM layer.

### 3.3. Tack Coat

Asphalt cements, emulsified asphalts, and cutback asphalts are commonly used as a tack coat to create bonding and share resistance between different asphalt layers [26]. Among them, the clean bond coat (CBC), which has the advantage of faster curing times and preventing any slippage between layers when comparing to traditional emulsions, has been widely used locally. In this study, such anionic asphalt emulsion (CBC), which was manufactured in McAsphalt, Toronto, Canada, was diluted with water at a rate of 1:1 in volume. The diluted CBC was then applied on the asphalt concrete surface at a rate of 0.5 L/m^2^. For instance, on a surface with a diameter of 15 cm, 9 mL of diluted tack coat was applied. The typical curing time ranges between 10 and 15 min, which is significantly shorter than conventional anionic slow-setting asphalt emulsion (SS-1).

### 3.4. Sample Preparation

The penetration test specimens were made in the shape of cylinders, with diameter of 150 mm and the height of 150 mm. Each specimen consists of a 5 cm height of SP 12.5, and 10 cm of SP 19. There are three types of specimens: control (CT) specimen without geogrid; interface specimen (IT) with geogrid located between SP 12.5 and SP 19; and middle specimen (MD) with geogrid located in the middle of SP 19. The interface specimen and middle specimen represent two common geogrid locations (i.e., between wearing course and binder course and in the middle of binder course). Figure 2 demonstrates the specimen size and location of geogrids for penetration test specimens.

**Figure 3:**
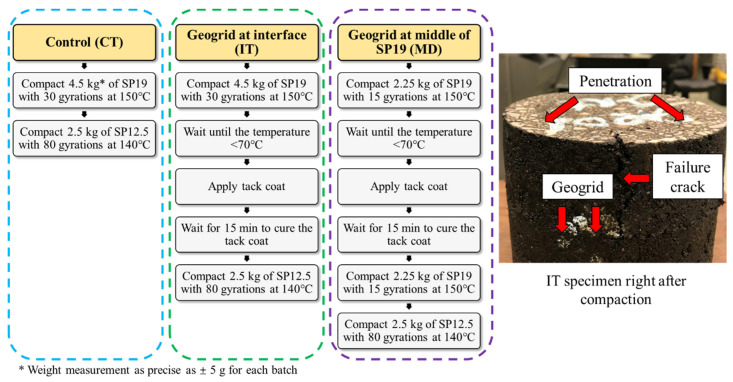
Process of specimen preparation.

The specimens were compacted using a Superpave gyratory compactor, manufactured in Gilson, Inc., Madison, WI, USA. After several trials, 4.5 kg of SP 19 and 2.5 kg of SP 12.5 in total were used for all specimens in order to ensure the consistent compaction effort. For every specimen, a total of 110 gyrations were applied. Compaction started with the binder course (SP 19). After the first layer of SP 19 compaction, the specimen was cooled down to have the surface temperature dropped to around 60 °C before applying with a diluted CBC tack coat. Then, the tack coat was completely cured for 15 min when it became dry and sticky. Consequently, the rest of the asphalt mixture will be compacted. Figure 3 provides a flow chart with the detailed compaction procedure for the three types of specimens. Such a procedure imitated the field pavement construction procedure and the sequence, and the base course and wearing course were paved in a layer-by-layer basis. The thickness of the base course and the wearing course used for the specimens were also commonly used locally. In addition, such a compaction sequence, asphalt layer depth, and the tack coat application were also used in a subsequent field project with geosynthetic reinforcement [8].

After compaction, all the specimens were ground on both sides to ensure the smoothness and obtain the final layer thickness for both SP 12.5 and SP 19. The bulk specific gravity (G_mb_) was tested as per AASHTO T 166 [27]. The average G_mm_ for the composite specimen was calculated to be 2.534, based on the G_mm_ of SP 19 and SP 12.5, as well as their weight proportions, see (6).

(6) $$G_{\mathrm{mm}}\left(\mathrm{composite}\right)=\frac{G_{\mathrm{mm}}\left(\mathrm{SP}\;19\right)\times \mathrm{weight}\;\mathrm{of}\;\mathrm{SP}\;19+G_{\mathrm{mm}}\left(\mathrm{SP}\;12.5\right)\times \mathrm{weight}\;\mathrm{of}\;\mathrm{SP}\;12.5}{\mathrm{weight}\;\mathrm{of}\;\mathrm{SP}\;19+\mathrm{weight}\;\mathrm{of}\;\mathrm{SP}\;12.5}\;=\frac{2.536\times 4.5\;\mathrm{kg}+2.529\times 2.5\;\mathrm{kg}}{7\;\mathrm{kg}}=2.534$$

Consequently, the voids in total mix (VTM) for each specimen can be calculated using (7) [27]. Details for the samples used for penetration test are listed in Table 3. For each sample including the control specimen, three replicate specimens were made. It should be noted that the calculation of G_mb_ did not take into account the effect of the geogrid. Nevertheless, specimens with a VTM outside the range of 7 ± 1% were discarded.

(7) $$\mathrm{VTM}=\left(1-\frac{G_{\mathrm{mb}}}{G_{\mathrm{mm}}}\right)\times 100\%$$

### 3.5. Test Set-Up

The current FN test protocol was developed and introduced in the AASHTO T 378 [28] as a simple test to evaluate the permanent deformation of asphalt mixture. Unlike the standard test, in this study, the diameter of cylinder was increased to 150 mm. In addition, the loading plate (top plate) was modified to have the smaller diameter of 100 mm. Therefore, during the test, the top plate penetrated into the specimen whereas the surrounding section with the geogrid will provide confinement. In such a case, the effects of geogrid reinforcement could be better investigated.

**Figure 4:**
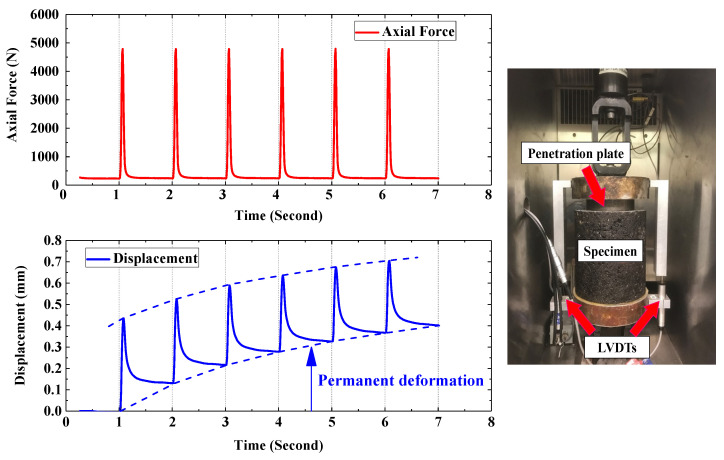
Axial force applied on specimen and its corresponding displacement (**left**); a view of specimen set up (**right**).

The test was conducted at a repeated compressive Haversine loading (one cycle consists of 0.1 s loading time and 0.9 s resting time) [14], using a MTS 810 loading frame which was manufactured in the USA. Two linear variable differential transformers (LVDTs) were attached on the frame to measure vertical deformation of contact area as a function of loading cycles, see Figure 4 for more details about the test set-up. Thus, the permanent deformation (rutting) was measured as the unrecoverable strain after each loading cycle.

The penetration test was conducted under a vertical stress ranging between 30 kPa and 600 kPa [28]. Based on the contact area (10 cm diameter circle), the vertical load ranged between 235 N and 4700 N. It should be noted that, since the aspect ratio of each cylindrical specimen was approximately 1:1. In order to minimize the horizontal frictions between specimen and the plates, latex films were greased and attached on both sides of each specimen before testing.

The stiffness of asphalt mixture changes significantly with the variation in temperature. A higher temperature will lead to the softening of asphalt binder, thus accelerating the rutting. In this study, such an effect of a high pavement surface temperature in warm seasons was simulated by setting the chamber temperature to 50 °C during both conditioning and testing. It is also in align with other rutting performance related tests such as FN test and wheel track test [1,3,15,21]. The test was set to terminate when the accumulated micro strain exceeded 40,000 με. At such conditions, the specimen surface was deformed significantly and macro cracks could be observed, indicating failure.

It should be noted that, in order to eliminate the boundary effects of the grid’s aperture, the diameter of loading plate was designed to be 100 mm, which is about four times the grid’s maximum aperture size. Due to the limitation of instrument for sample preparation and testing, the diameter for the cylindrical specimen was 150 mm; therefore, the maximum distance between the edge of the loading plate and the edge of specimen was 2.5 cm. In the next phase of the research, specimen size will be further increased to provide extra confinement and to eliminate the boundary effects on the specimen, since it was found that the tension and bulging usually occur within 2 to 3 cm from the edge of tire/loading plate [16,19,29].

## 4. Results and Discussion

### 4.1. Development of Permanent Deformation

**Figure 5:**
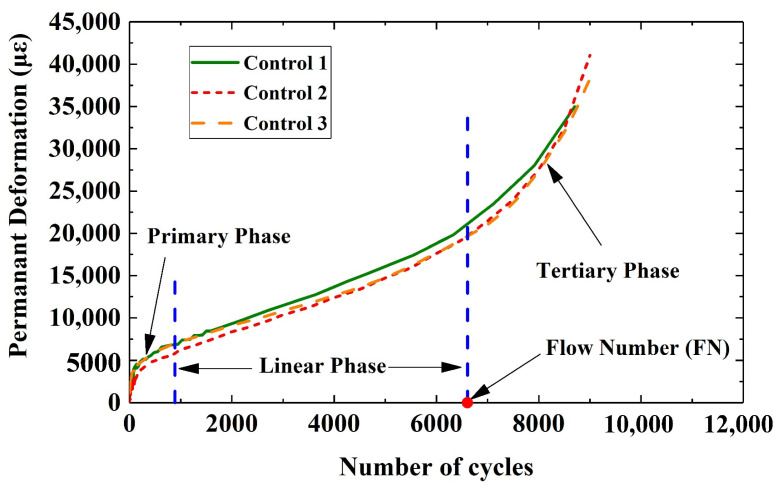
Development of permanent deformation of control specimen subjected to cyclic loading.

Figure 5 shows the evolution of permanent deformation at the centre of a representative control sample subjected to penetration. It could be seen from Figure 5 that the trend of the deformation under penetration develops in a similar form compared to those in wheel track test [2,3,10], as well as the original flow number (FN) test [14,15]. Typically, three phases could be easily distinguished. It should also be noted that, during the creep test, ordinary concrete exhibits similar three-stage properties on the relationship between the strain and the time [30,31,32].

**Figure 6:**
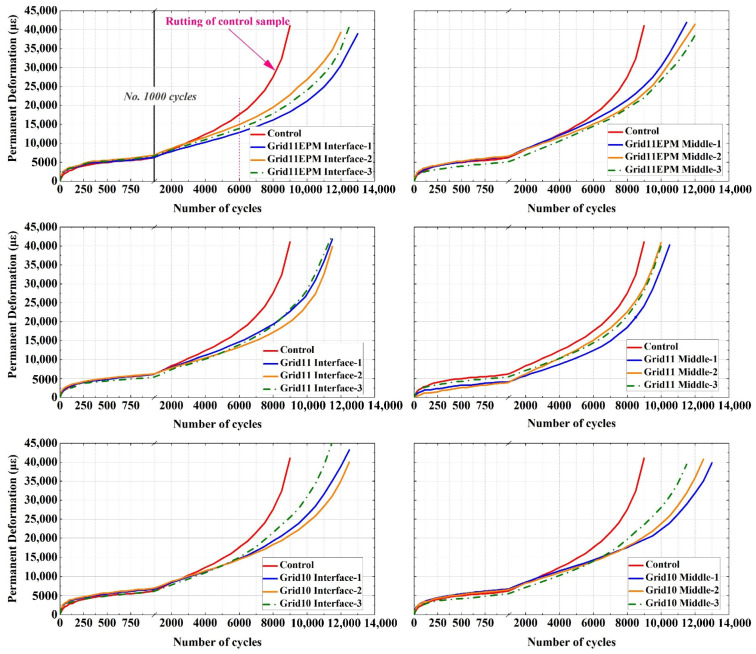
Development of permanent deformation of control and geogrid-reinforced asphalt samples.

Furthermore, the relationships between permanent deformation and the number of cycles for geogrid-reinforced asphalt samples are summarized for comparison with the control specimen. The development of permanent deformation for control and Grid11EPM-treated asphalt samples are shown in Figure 6. The number of samples were presented in the two-step scale to better present the variation between No. 1 and No. 1000 cycles. After No. 1000, results were drawn on a larger scale. It should be noted that, in order to clearly present the effects of the geogrid, only one of the three curves for the control specimen was drawn. Such a curve was used to be compared with all the other specimen types to ensure consistency. In fact, results from the three curves for control specimens were highly resembled and overlapped. The standard deviations of the curves between each specimen accounted to be less than 0.20.

For each reinforced-asphalt mix, the results of the three parallel specimens were shown. It was evident from the figures that, at the initial period (<3000 cycles), the trends of deformation development of the control and geogrid-reinforced specimens were quite similar. This phenomenon can be physically explained that as soon as the load was applied on the specimen, the aggregates were slightly rearranged and the mixture hardened [18], whereas the geogrid had limited effects on this rearrangement and hardening.

However, with the increase in loading cycles, geogrid-reinforced specimens tended to have a more distinct impact on load distribution, resulting in slower rutting progressing, compared to the control specimen. In addition, such a reinforcement significantly extended the life of treated asphalt specimens before failure. The control specimen reached 40,000 με at approximately 9000 cycles. In contrast, to reach the same level of permanent deformation, the geogrid-reinforced asphalt could withstand approximately 10,000 to 12,000 cycles. In other words, under the same number of cycles, the maximum performant deformation was decreased compared to the control specimen [16]. Thus, it also indicated that geogrids will indeed contribute to the resistance of permanent deformation in geogrid-reinforced pavements. Such a phenomenon coincides with the findings of past studies [1,10]. Among the three types of geogrids, the Grid11EPM and the Grid10 treated specimens tended to have relatively longer service life before entering the tertiary phase, compared to Grid11 treated specimens.

**Figure 7:**
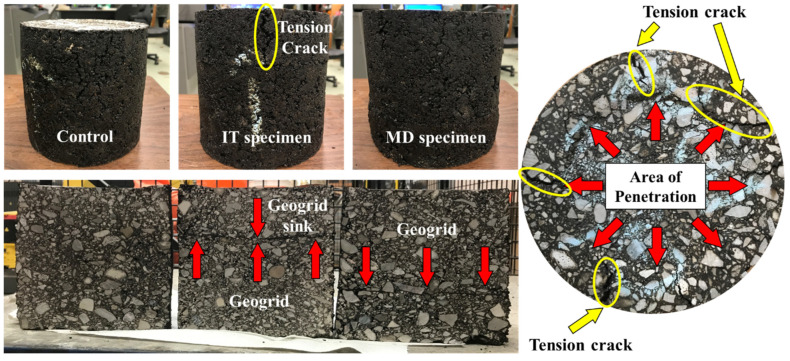
Comparisons of specimens after penetration test (**left**), top surface of specimen after penetration (**right**). Note the change in the location of the geogrid due to loading. In addition, yellow ovals circulate tension cracks developed during penetration.

Additionally, Figure 7 illustrates the top-down cracks (TDCs) and the cross-section of specimens after testing. The penetration in the centre of specimen (rutting) created tension and shear stress in the surrounding area, thus causing the cracks to emerge near the penetration edge. The cracks then developed and propagated downwards due to repeated loading forming the TDCs [33,34]. Figure 7 further indicated that, after penetration, the middle of geogrid in IT specimens had been deformed downwards. However, there is no noticeable gap between the geogrid and the asphalt mixture on either side, indicating good bonding during the test. In addition, the cracks initiated from the top of the asphalt surface were found to be either cut off or forced to turn laterally and move along the interface. Thus, such reinforced layer may contribute to the structural capacity of the pavement [12,34]. Furthermore, no distinct cracks were observed beneath the geogrid indicating that both tension and shear were reduced in the location beneath geogrid. Such effects were more distinct in IT specimens since the geogrid is near to the surface. However, it was also found that the width of TDCs near the surface of IT specimens was wider than those in CT and MD specimens.

Overall, the development of permanent deformation and the strain rates in both control and reinforced specimens under penetration tests were similar to those in other rutting performance tests [1,3,15]. It can also be noted that with the geogrid reinforcement, the linear phase within the permanent deformation development was considerably extended, especially in Grid11EPM and Grid10 treated samples. In addition, the failure cracks were prevented from further propagation owing to the geogrid segregation. Such findings denoted an improvement of rutting resistance under normal service life in reinforced samples.

### 4.2. Fitting of Permanent Deformation

**Figure 8:**
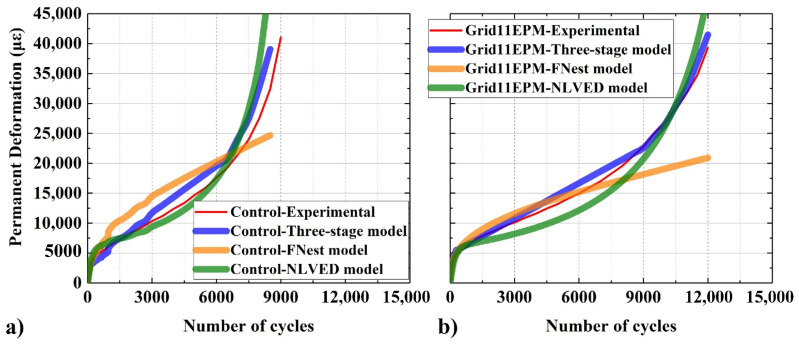
Illustration of different fitting methods, for control (**a**), and Grid11EPM reinforced specimens (**b**).

In this section, the three selected models (Equations (3)–(5)) were used to fit the experimental data from the penetration test. Equation (3) was selected due to the fact that such a three-stage model conforms to the original model without being simplified as in Equations (1) and (2). Equations (4) and (5) provide further insight due to their reliance on different mechanisms of the test. Table 4 summarizes the fitting results of permanent deformation based on the three-stage model, FNest model, and nonlinear viscoelastic (NLVED) model. It could be seen from the figure that the three-stage and NLVED models had high levels of coefficients of determination (R^2^), greater than 0.90, indicating good fitting properties. Moreover, Figure 8 presents the typical fitting curves for control and geogrid-reinforced asphalt, between different fitting methods. In terms of fitting, it was evident that all the three methods were able to satisfactorily predict the primary phase of deformation development. Specifically, the three-stage and NLVED fitting curves both exhibited the clear three stages of deformation development, while the FNest was not able to have a close to nature fitting for the linear and tertiary phase, compared to the experimental results. Regarding the fitting difficulties, the regression operation for three-stage model was relatively easier than NLVED since the regression was divided into simple steps and with fewer regression coefficients to calculate.

Overall, both of three-stage and NLVED models showed similar regression results. They could both be used for the fitting and prediction of rutting of control and geogrid-reinforced asphalt mixtures.

### 4.3. Strain Rate

**Figure 9:**
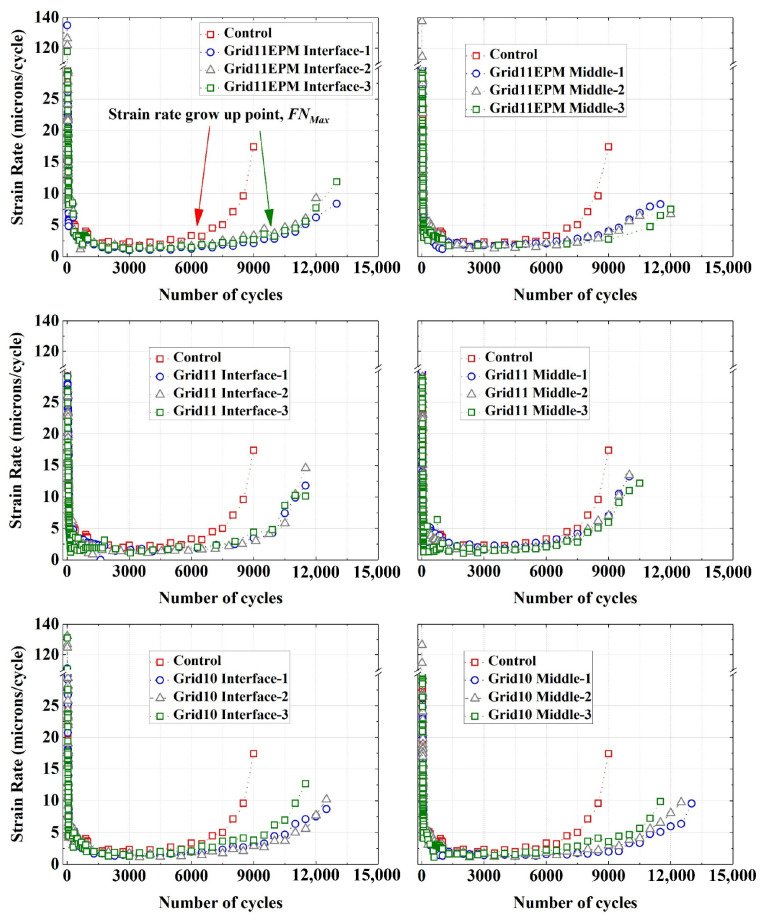
Strain rate of control and geogrid-reinforced asphalt samples.

Before calculating the FN, the strain rate of permanent deformation is usually calculated. To minimize the noise, a moving average recalculation was used in this study (8) to smooth the strain rate curve. As recommended in previous studies [15,24], a moving average period (MAP) equal to 5 was adopted. The strain rates for different samples were summarized in Figure 9. It should be noted that the ordinate of permanent deformation has been modified and there is a break in the scale from 30 to 110. In addition, the values below 30 were drawn in a magnified scale so that the strain rates after 5000 are much more recognizable.

(8) $$\frac{d{\left(\varepsilon_{p}\right)}_{i}^{’}}{dN}=\frac{1}{5}\left[\frac{d{\left(\varepsilon_{p}\right)}_{i-2\Delta N}}{dN}+\frac{d{\left(\varepsilon_{p}\right)}_{i-\Delta N}}{dN}+\frac{d{\left(\varepsilon_{p}\right)}_{i}}{dN}+\frac{d{\left(\varepsilon_{p}\right)}_{i+\Delta N}}{dN}+\frac{d{\left(\varepsilon_{p}\right)}_{i+2\Delta N}}{dN}\right]$$

where, $\frac{d{\left(\varepsilon_{p}\right)}_{i}^{’}}{dN}$ = smoothened strain rate at load cycle (microns/cycle), *i*;
*∆N* = data sampling interval.

Initially after loading, during Stage I (the primary phase), significantly higher strain rates were found in both control and geogrid-reinforced specimens, compared to the rates in the later stages. Overall, there were no considerable differences between the control and geogrid-reinforced specimens, regarding strain rate, during the Stage I (the primary phase). However, the rate dropped sharply within the first 500 cycles, representing entering the Stage II (the linear phase). Within Stage II, both the control and geogrid-reinforced specimens exhibited constant and very low strain rates, ranging between 0 and 5 microns/cycle. Nevertheless, the constant rate remained for a greater number of cycles in reinforced specimens than control specimens, indicating that reinforcement could delay entering Stage III (the tertiary phase). Moreover, the trend of stain rate remained approximately identical for the three parallel specimens of each geogrid-reinforced sample, although their development of deformation was slightly different, as they are presented in Figure 6, demonstrating successful data acquisition. Consequently, it can be concluded that the geogrid reinforcement contributed to a more sustainable resistance against permanent deformation in asphalt mixtures.

### 4.4. Flow Number

As it was illustrated previously, there are multiple methods to quantify the FN value. However, those values resulting from different methods could differ significantly from each other [15]. This was also evident from the results based on fittings for geogrid-reinforced asphalt, see Table 4. Theoretically, the FN value indicates the timing when the deformation starts to increase significantly faster. The traditional rate-based solution tried to find the minimum strain rate. However, as is seen in Figure 9 and indicated by other studies, due to the scattering in data acquisition, multiple points shared the same minimum strain rate value [18]. In that case, some literature suggested using the first point that corresponds to the minimum strain rate [24,25]. Under that scenario, the calculated FN could fall in the beginning or middle of linear phase, so the definition of FN could not be accurately achieved. Therefore, based on the results and the condition of this study, a more realistic FN calculation was proposed in (9).

$$FN_{Max}=Max\;\left(N_{k}\right),\;N_{k}>N_{I}$$

(9) $$\frac{d{\left(\varepsilon_{p}\right)}_{k}^{’}}{dN}=3$$

where $\frac{d{\left(\varepsilon_{p}\right)}_{k}^{’}}{dN}$ = smoothened strain rate at load cycle, *k*.

It should be noted that such a calculation was based on the acquisition of strain rate. It could be either from experimental results, which is the case in this paper, or from fitting algorithms, such as FNest and NLVED models. The FN obtained from (9) is called *FN_Max_*, since it indicates the maximum number of cycles that remain at a low strain rate (<3) and the *FN_Max_* must be higher than *N_I_*. This value is in line with the principle of FN definition and coincides with the timing when the asphalt mixture enters the tertiary phase.

**Figure 10:**
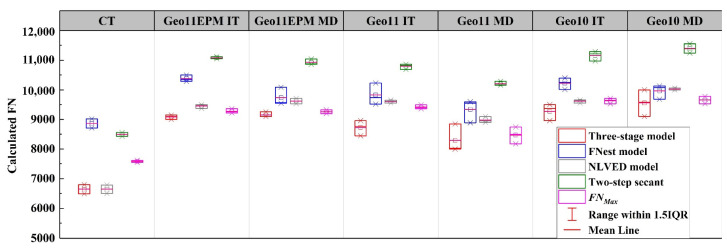
Calculated FN based on different prediction methods.

Figure 10 summarizes the FN values obtained from different fitting models. In this research, Liu’s method [15] was followed to acquire the FN based on two-step secant method. In terms of different models, the FNest and two-step secant model exhibited obviously higher FN values than the other methods. The FNest, as indicated previously, could not accurately fit the deformation after the linear phase. Due to the nature of two-step secant method, its predicted FN was always located at the tertiary phase. Therefore, FN predicted by such a method is doomed to be larger than other methods [15]. Such a phenomenon was confirmed in this study.

Overall, NLVED, two-step secant, and *FN_Max_* methods had more consistent prediction among the three replicates. The FN values predicted by NLVED method were very close to *FN_Max_* values for the geogrid-reinforced asphalt. To the author’s best knowledge, the two methods mentioned above have better FN predictions than other mathematical methods. In particular, the *FN_Max_* method may be more practical given its calculation principle and its simplicity. It should be also noted that previous studies shared a similar principle to find the FN value, that is, to find the number of cycles that corresponds to the time point when its relationship with permanent deformation start deviating from a straight line [18]. However, the calculation method for *FN_Max_* is convenient and reproducible and it was successfully used to quantitatively evaluate the effects of different geogrids on the rutting resistance of asphalt mixture.

### 4.5. Reinforcement Location and Types of Geogrids

**Figure 11:**
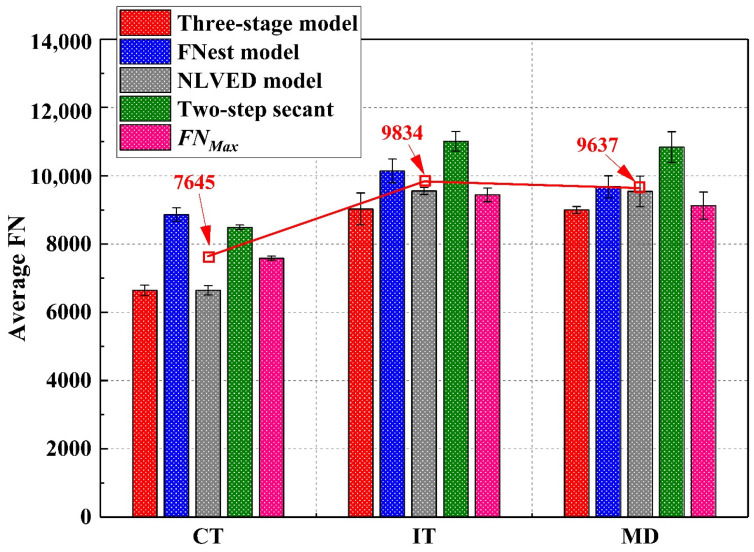
Differences between IT and MD specimens.

Figure 11 summarizes the differences for specimens reinforced with geogrids at different locations. First of all, a significant growth of FN was shown between control samples and geogrid-reinforced samples. The average FN of the CT sample, including all prediction methods, accounted for 7645, whereas the IT samples and MD samples had average FN values making up 9834 and 9637, respectively, accounting for a 26% to 29% increase due to the geogrid reinforcement. In addition, there is a small increase in FN in the IT samples from MD specimens. However, past field trials suggested a deeper geogrid location may decrease the shear strength as well as prevent slipping cracks [6,35]. In this regard, a geogrid embedded in the MD location will be preferred for a thick asphalt binder course.

**Figure 12:**
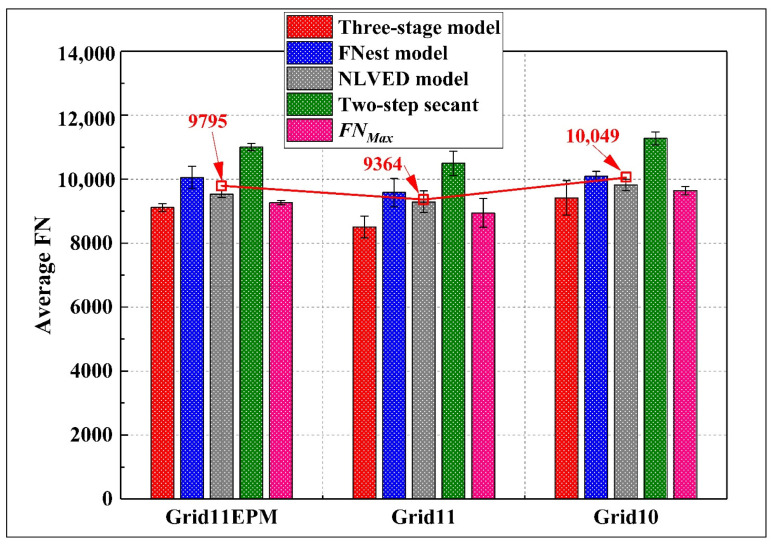
Differences between various types of geogrids.

In terms of the geogrid type, it was suggested that the Grid10 with a smaller aperture size had slightly better performance regarding the rutting resistance compared to the other two geogrid types, as seen in Figure 12. Although the three grids themselves had the same tensile strength, a smaller apertured grid could provide larger contact area with the aggregates, thus contributing to more distributed stress in the pavement structure. On the other hand, Grid11EPM performed better than Grid11 regarding rutting performance; the reason could be due to the EPM, which melted during compaction and created addition cohesion between asphalt mixture and the grid. In that case, the shear strength of the structure was improved.

It should be noted that this observed increase in FN only considered one specific asphalt mixture type using this novel penetration test. The effects and principles of geogrid reinforcement on the rutting performance will be further investigated by a long-term field construction and instrumentation project.

## 5. Conclusions

Overall, this paper presented a preliminary study on the rutting performance of geogrid-reinforced asphalt mixture. A penetration test was proposed to simulate and investigate the rutting performance in geogrid-reinforced asphalt. In addition, a new FN calculation was defined and used for evaluation among different geogrid types. The test and data analysis method presented in this study could be an important reference for future investigations on geosynthetic-reinforced pavement. Based on the laboratory tests and analyses of the data, the following conclusions and discussions can be drawn:

(1) The proposed penetration test is a convenient experimental method to measure the rutting performance of geogrid-reinforced asphalt mixtures. Under penetration, the performance deformation followed the three-stage phases. In comparison, the geogrid-reinforced asphalt samples entered the tertiary phase later than those of control samples.
(2) The top-down cracks (TDCs) generated by repeated loading were prevented from propagating deeper due to the presence of the geogrid reinforcement. Such phenomenon is more distinct when the geogrid was put at the interface between wearing and binder course.
(3) Among the current fitting models for permanent deformation, the three-stage and NLVED models showed better fitting results compared to the experimental data with higher R^2^ values. The regression operation for three-stage model was more convenient since the regression was divided into steps and fewer regression coefficients were required to calculate.
(4) The strain rate dropped sharply in the first 500 cycles for both control and geogrid reinforced samples. After 500 cycles, the strain rate remained relatively constant and at very low level, ranging between 0 and 5 microns/cycle. Nevertheless, the constant rate was extended for a greater number of cycles in reinforced specimens than control specimen indicating that the geogrid could delay entering the tertiary phase.
(5) Based on the strain rate, a new FN calculation was proposed, which is called *FN_Max_*. *FN_Max_* denoted the maximum number of cycles, which still remains at a low strain rate (<3). FN valued predicted by the NLVED and *FN_Max_* methods were similar to each other and were more consistent. In particular, the *FN_Max_* method could be more practical given its calculation principle and its simplicity.
(6) An average FN increase between 26% and 29% was observed for geogrid-reinforced asphalt compared to control samples. In addition, there is a small increase in FN in IT samples from MD specimens. On the other hand, Grid10 with smaller aperture size had slightly better rutting resistance compared to the other two geogrid types, followed by Grid11EPM and Grid11.

It should be noted that some limitations exist, including the specimen size and the confinement. Therefore, in the next phase of research, more asphalt types and geogrid types will be incorporated to validate and further develop the laboratory investigation.

## Figures and Tables

**Table 1:** Summary of recent models for permanent deformation based on standard FN test.

Source	Flow Number (FN) Model	Particular Variable	Equation
[3]	$\varepsilon_{p}=a\left(N^{b}+e^{cN}\right)$	a, b, c material constant, obtained by regression	(1)
[22]	$\varepsilon_{p}=aN^{b}+d\left(e^{cN}-1\right)$	a,b,c,d material constant, obtained by regression	(2)
[23]	Stage I:	a, b, c, d,f material constant, obtained by regression	(3)
	$\varepsilon_{p}=aN^{b}$		
	Stage II:		
	$\varepsilon_{p}=\varepsilon_{I}+c\left(N-N_{I}\right)$		
	Stage III:		
	$\varepsilon_{p}=\varepsilon_{\mathrm{II}}+d\left(e^{f\left(N-N_{\mathrm{II}}\right)}-1\right)$		
[24]	$\varepsilon_{p}=\frac{1}{\beta }{\left[-\ln\left(1-\frac{N}{\gamma }\right)\right]}^{1/\alpha }$	α, β, γ regression parameter. *FNest* flow number; equals to *N_II_*	(4)
	$FNest=\gamma \left[1-\exp\left(\frac{1}{\alpha }-1\right)\right]$		
[21]	$\varepsilon_{NLV}=\frac{\sigma_{0}\left(1-e^{-BNt_{0}}\right)}{2AB\left(\frac{t_{0}^{2}B^{2}}{4\pi^{2}}+1\right)}$	*ε_NLV_* permanent viscous strain. *ε_NLVED_* permanent viscous strainincorporating damage variable. *σ*_0_ peak value of the haversine load. *t*_0_ loading time of a cycle. *A,B* material constant *D* Chaboche and Lemaitre damage variable	(5)
	$\varepsilon_{NLVED}=\frac{\sigma_{0}\left(1-e^{-BNt_{0}}\right)}{\left(1-D\right)2AB\left(\frac{t_{0}^{2}B^{2}}{4\pi^{2}}+1\right)}$		

**Table 2:** Physical properties for three types of fiberglass geogrids.

Category	Unit	Grid11EPM	Grid11	Grid10
Ultimate tensile strength	kN/m	100.0	100.0	100.0
Strain at ultimate tensile strength	%	<3	<3	<3
Tensile strength at 2% strain	kN/m	75.0	80.0	80.0
Secant stiffness at 2% strain	kN/m	3750	4000	4000
Aperture size	mm	25.4	25.4	12.7
Melting point of coating	°C	400	400	400
Melting point of glass	°C	820	820	820
Melting point of EPM	°C	124	--	--
Mass/Unit area	g/m^2^	432	420	420

**Table 3:** Results of bulk specific gravity and air void ratio of the specimens.

Specimen Label	G_mm_	G_mb_	VTM (%)
Control (CT)	2.534	2.354~2.410	7.02~7.10
Geo11EPM Interface (IT)	2.534	2.330~2.365	6.67~8.05
Geo11EPM Middle (MD)	2.534	2.341~2.367	6.59~7.62
Geo11 Interface (IT)	2.534	2.367~2.368	6.55~6.59
Geo11 Middle (MD)	2.534	2.370~2.373	6.35~6.47
Geo10 Interface (IT)	2.534	2.358~2.367	6.59~6.95
Geo10 Middle (MD)	2.534	2.348~2.362	6.79~7.34

**Table 4:** Summarize of fitting for permanent deformation of asphalt samples under penetration test.

	No. of Specimen	Three-Stage Model (Equation (3))				FNest Model (Equation (4))			Nonlinear Viscoelastic (NLVED) Model (Equation (5))		
		R^2^	*N_I_*	*N_II_*/FN	Ave. SR	R^2^	FN	Ave. SR	R^2^	FN	Ave. SR
CT	1	0.98	169	6799	±0.23	0.73	9019	±1.59	0.97	6500	±0.40
	2	0.98	189	6489	±0.36	0.75	8709	±1.45	0.95	6789	±0.54
Geo11EPM IT	1	0.98	299	9099	±0.25	0.83	10,348	±1.24	0.95	9369	±0.49
	2	0.99	349	8999	±0.22	0.83	10,497	±1.47	0.96	9499	±0.56
	3	0.97	399	9147	±0.29	0.87	10,285	±1.54	0.98	9479	±0.52
Geo11EPM MD	1	0.99	269	9257	±0.30	0.84	9544	±1.54	0.92	9528	±0.55
	2	0.94	229	9099	±0.26	0.83	10,083	±1.50	0.97	9619	±0.51
	3	0.96	255	9099	±0.21	0.80	9567	±1.20	0.94	9709	±0.49
Geo11 IT	1	0.97	345	8958	±0.18	0.82	9732	±1.40	0.94	9649	±0.59
	2	0.95	296	8448	±0.14	0.81	10,227	±1.50	0.98	9549	±0.62
	3	0.93	286	8768	±0.21	0.78	9516	±1.70	0.96	9606	±0.48
Geo11 MD	1	0.98	256	8847	±0.28	0.78	8886	±1.61	0.97	9098	±0.44
	2	0.96	199	7999	±0.20	0.85	9544	±1.43	0.99	8950	±0.50
	3	0.95	399	8028	±0.22	0.80	9594	±2.02	0.93	8899	±0.40
Geo10 IT	1	0.96	237	9369	±0.32	0.82	10,253	±1.56	0.94	9639	±0.55
	2	0.96	269	9499	±0.30	0.74	10,394	±1.50	0.97	9549	±0.62
	3	0.95	369	8959	±0.22	0.72	10,012	±0.46	0.93	9655	±0.51
Geo10 MD	1	0.94	305	9579	±0.30	0.85	10,129	±1.24	0.92	9998	±0.41
	2	0.96	269	9999	±0.26	0.73	10,076	±1.50	0.97	10,059	±0.59
	3	0.99	257	9099	±0.28	0.83	9681	±1.38	0.97	10,028	±0.44

Ave. SR: Average standard residuals.

## Data Availability

Data will be made available on request.
